# Supplementary material for: Towards unified AI-driven fracture mechanics: the extended deep energy method (XDEM)
Source: Nat Commun. 2026 Aug 15;17:8492. doi: 10.1038/s41467-026-76748-1 (PMC13484471; doi:10.1038/s41467-026-76748-1)
Supplement: Supplementary file 3 [file 41467_2026_76748_MOESM3_ESM.zip › pic/Results/Crack_propagation/inclusion/Figure_5.pdf]

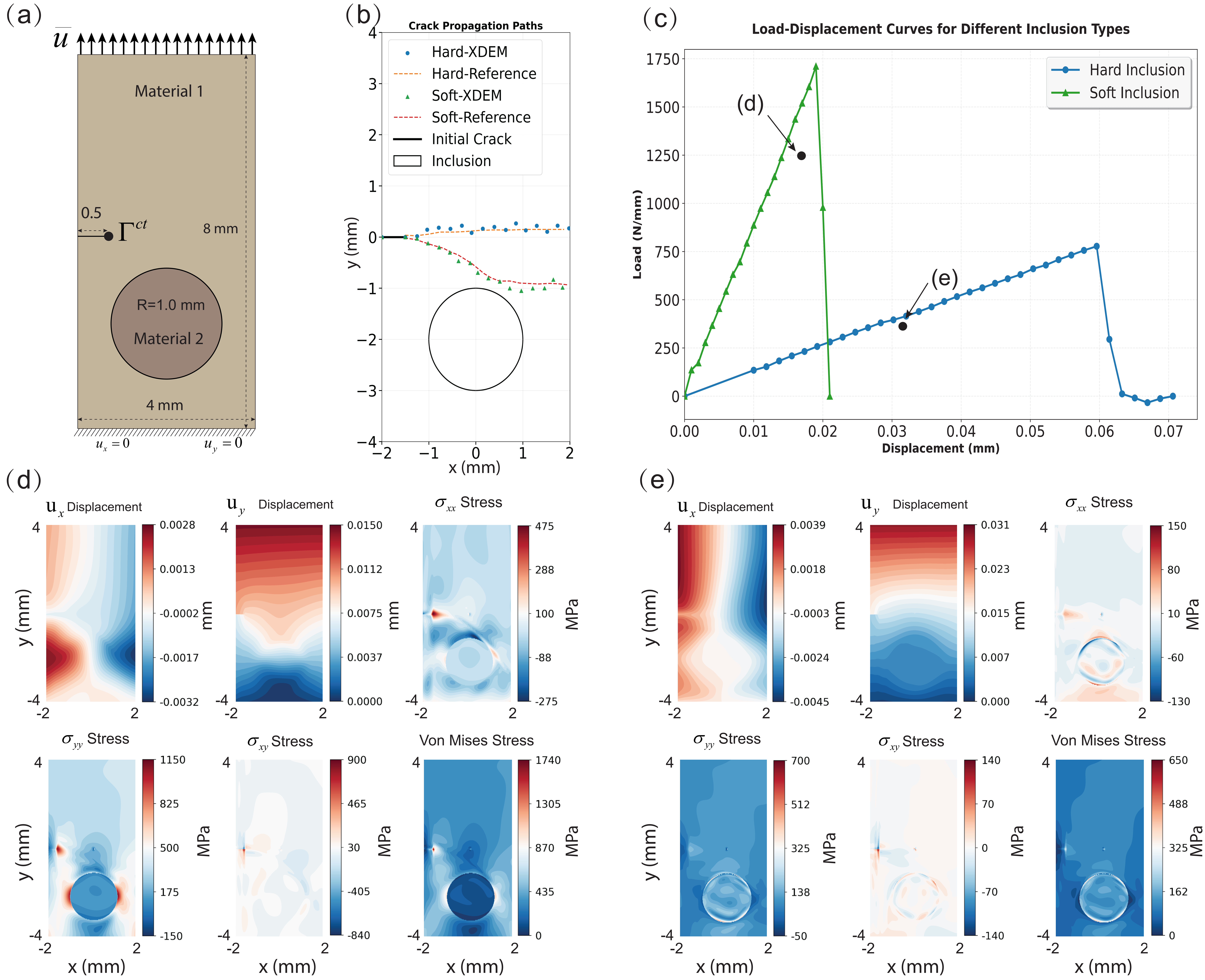
(a)
Material 1
0.5
Γ^{ct}
8 mm
R=1.0 mm
Material 2
4 mm
$u_x = 0$
$u_y = 0$
(b)
Crack Propagation Paths
Hard-XDEM
Hard-Reference
Soft-XDEM
Soft-Reference
Initial Crack
Inclusion
y (mm)
x (mm)
(c)
Load-Displacement Curves for Different Inclusion Types
Hard Inclusion
Soft Inclusion
Load (N/mm)
Displacement (mm)
(d)
(e)
u_x Displacement
u_y Displacement
σ_{xx} Stress
σ_{yy} Stress
σ_{xy} Stress
Von Mises Stress
mm
MPa
